# Direct access valve-in-valve implantation for management of complex valvulopathy

**DOI:** 10.1002/ccd.28179

**Published:** 2019-04-08

**Authors:** Alexander P. Kossar, Michael Borger, Isaac George

**Affiliations:** 1 Division of Cardiothoracic Surgery, New York Presbyterian Hospital–Columbia University Medical Center, New York, New York; 2 Department of Cardiac Surgery, Leipzig Heart Center, Leipzig, Germany

**Keywords:** aortic valve disease (AVD), interventional devices/innovation (IDI), mitral valve disease (MVD), structural heart disease intervention (SHDI), surgery, transcatheter valve implantation (TVI), valvular (SVAL)

## Abstract

The management of bioprosthetic structural valve degeneration requires complex surgical or transcatheter re-intervention for which many high-risk patients are not considered candidates. Here, we describe a technique for a direct surgical access valve-in-valve implantation in patients with complex bioprosthetic valvulopathy for whom standard surgical valve replacement and percutaneous interventions were high-risk and contraindicated, respectively.

## 1 | INTRODUCTION

Bioprosthetic heart valves (BHV) are subject to increased susceptibility to structural valve degeneration (SVD) over time. Transcatheter techniques such as transcatheter aortic valve replacement (TAVR) or valve-in-valve (VIV) implantation are currently options for both aortic and mitral valve SVD in patients with prohibitive surgical risk for reoperative valve surgery, but are contraindicated when either risk of coronary obstruction or left ventricular outflow tract obstruction exist.^1–5^ In such patients, direct surgical VIV implantation has emerged as an alternative method of catheter-based valve intervention.^6^ Here, we describe our technique for elective direct surgical access VIV implantation in high-risk patients with severe aortic and mitral SVD, respectively, not otherwise amenable to standard transcatheter management.

## 2 | CASE SERIES

### 2.1 | Case 1

**Figure 1:**
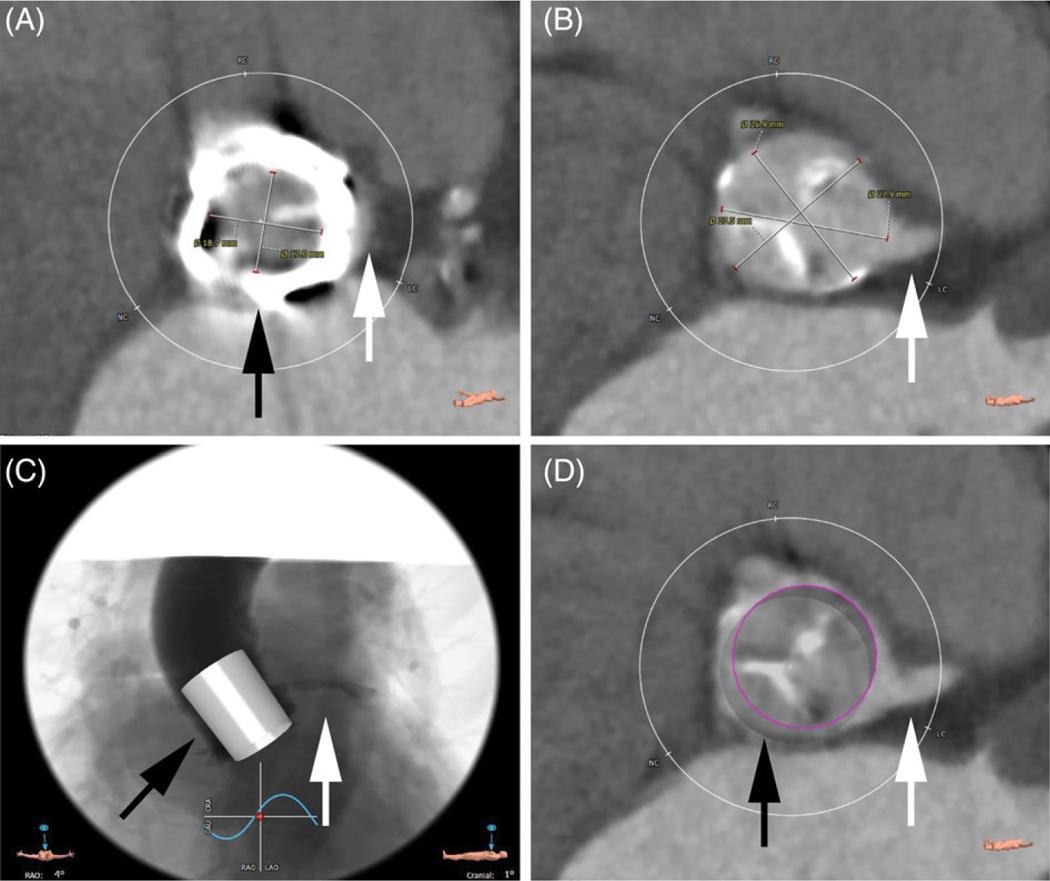
Preoperative CT modeling for case 1. A, Surgical valve frame (black arrow) in relation to aortic sinus (white arrow). B, Left coronary ostium (white arrow). C and D, Proposed virtual valve (black arrows) in relation to left coronary ostium (white arrows)

A 75-year-old woman status-post surgical aortic valve replacement (AVR) with a #21 LivaNova Sorin Mitroflow bioprosthesis (London, UK) 7 years prior, referred for management of bioprosthetic aortic stenosis (AS). Transesophageal echocardiogram (TEE) revealed a reduced left ventricular ejection fraction (LVEF) of 35%, severe AS and insufficiency (AI), severe mitral regurgitation (MR) with mild dilatation of the mitral valve annulus, and a 3 cm left atrial appendage thrombus (see Supporting Information Video 1). Her Society of Thoracic Surgeons (STS) risk score was calculated at 9.9% for isolated AVR. Computed tomography (CT) of the chest showed a profoundly calcified aortic BHV embedded within the aortic wall and coronary ostia. Surgical AVR would have necessitated complex aortic root revision and, given the patient’s age, medical co-morbidities, and calcified coronary ostia, we planned a surgical VIV implantation using a transcatheter valve. Following redo sternotomy, initiation of cardiopulmonary bypass (CPB), endocardial CryoMAZE procedure, and left atrial appendage ligation, a mitral valve repair with a #30 Carpentier-Edwards Physio II ring (Irvine, CA) was performed. Oblique aortotomy revealed findings consistent with the CT scan. A VIV procedure was predicted to provide adequate hemodynamics after valve excision (Figure 1). The BHV leaflets and posts were excised, and a #23 Medtronic Evolut valve (Minneapolis, MN) was deployed within the prior sewing ring, positioned such that the bottom of the Evolut frame was 2 mm below the surgical valve sewing ring (see Supporting Information Video 2). The coronary ostia were subsequently noted to be patent and the aorta was patched with bovine pericardium. Finally, the tricuspid valve was repaired using a #28 Carpentier-Edwards MC3 ring (Irvine, CA).

The patient was weaned from CPB, and TEE demonstrated excellent valvular function without leak. The patient was extubated on postoperative day (POD) #2 and had a permanent pacemaker placed on POD#10 for a prolonged sinoatrial pause. There was no evidence of AI or AS, mean gradient was 12 mmHg, and all aortic dimensions were within normal limits on transthoracic echocardiogram from POD#11. The patient was discharged to a rehabilitation facility on POD#14 and was doing well at 3 year follow-up without increase in valve gradients (see Supporting Information Video 3).

### 2.2 | Case 2

**Figure 2:**
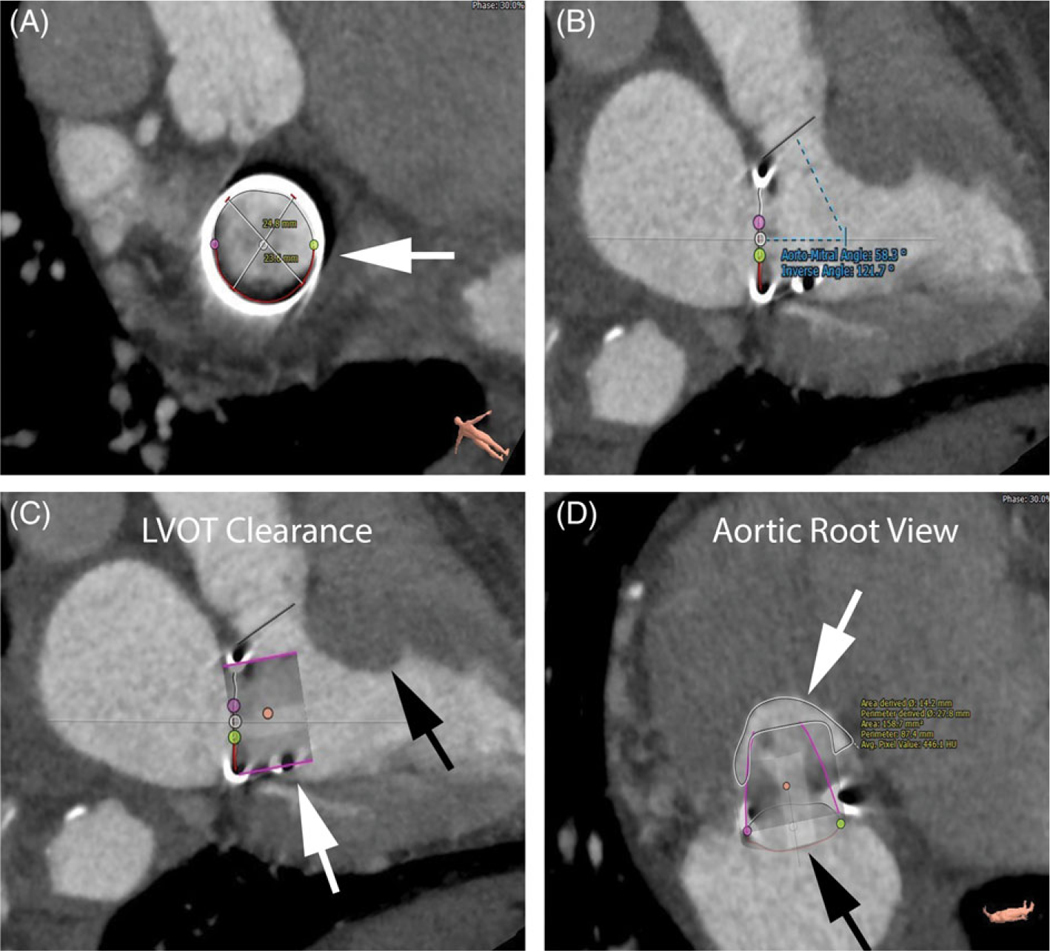
Preoperative CT modeling for case 2. A, Surgical valve frame (white arrow). B, Aorto-mitral angle. C, Virtual valve (white arrow) in relation to intraventricular septum (black arrow) demonstrating poor LVOT clearance. D, View of aortic root with virtual valve (black arrow) and calculated neoLVOT area (white arrow)

**Figure 3:**
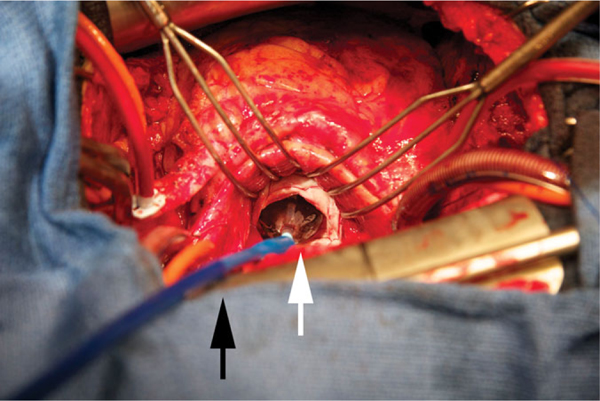
Surgical valve-in-valve implantation with Sapien 3 delivery system (black arrow) and valve frame (white arrow) for a severe bioprosthetic mitral insufficiency

An 84-year-old woman status-post surgical mitral valve replacement with a #27 Medtronic Hancock II (Minneapolis, MN) bioprosthesis 12 years prior, referred for management of severe MR. TEE revealed a small left ventricular outflow tract (LVOT) and large bioprosthetic struts. Due to concern for LVOT obstruction which precluded a full transseptal mitral VIV procedure, in conjunction with elevated surgical risk (Society of Thoracic Surgeons risk score of 13.2% for mitral valve replacement), direct surgical VIV implantation was performed (Figure 2). Following redo sternotomy and initiation of CBP, a left atriotomy revealed a mitral BHV with tears in 2 of 3 leaflets. All leaflets were excised, and a #26 Edwards Lifesciences Sapien XT (Irvine, CA) valve was inserted through the atriotomy and deployed within the prior valve frame with adequate LVOT clearance, positioning the atrial end of the Sapien XT frame just at the base of the sewing ring of the bioprosthetic valve with –1 mm left atrial protrusion (Figure 3). Given the height of the surgical valve (19 mm) and height of a fully expanded #26 Sapien XT, this ensured no additional ventricular protrusion of the Sapien XT valve beyond the surgical valve struts. Gentle atrial retraction was applied to the valve during deployment to optimize LVOT clearance and achieve the proper position. The patient was weaned from CBP without incident, and TEE revealed a trace, hemodynamically-insignificant paravalvular leak.

Shortly after admission to the ICU, increasing sanguineous chest tube output and vasopressor requirements prompted return to the OR for re-exploration. The patient was placed on CBP and an inferolateral left ventricular wall perforation was identified and directly repaired. This injury was likely due to balloon nose cone perforation of the Sapien XT valve balloon, as deployment was performed without wire access. TEE on POD#1 was negative for bioprosthesis dysfunction, and the patient was extubated on POD#2. Her postoperative course was complicated by atrial fibrillation. She was discharged to home on POD#9 and was doing well at follow-up (see Supporting Information Video 4).

## 3 | DISCUSSION

Here, we present a strategy for elective management of bioprosthetic valvulopathy in high-risk patients who are poor candidates for standard transcatheter therapy. In case 1, the aortic BHV was embedded within the aortic wall and coronary ostia which, if replaced surgically, likely would have subjected our high-risk patient to a complex aortic root procedure. In case 2, the patient’s LVOT and BHV strut dimensions would have increased the risk of LVOT obstruction following standard transcatheter management.

Direct surgical VIV implantation offers several potential benefits for a select group of patients requiring BHV replacement not amenable to percutaneous intervention. In patients with anatomical or technical issues precluding BHV explanation, direct VIV implantation with preservation of the original bioprosthesis annulus can limit intra-operative cardiac tissue trauma, and limit operative times. Preservation of the original bioprosthesis annulus may also decrease the risk of developing significant paravalvular leak when compared to standard surgical replacement. This technique has intrinsic limitations, the main one being the risk of prosthesis-patient mismatch and unknown long-term durability, which is dependent on the transcatheter valve. An additional limitation is the potential requirement for postoperative anticoagulation.^7^

## 4 | CONCLUSION

In high-risk patients with bioprosthetic SVD for whom surgical valve replacement is indicated and for whom standard percutaneous therapies are contraindicated, direct access VIV implantation is an alternative approach that may reduce morbidity associated with complex anatomy.

## Supplementary Material

Video 1

Video 2

Video 3

Video 4

Video Legends
