# Supplementary material for: Direct access valve-in-valve implantation for management of complex valvulopathy
Source: Catheter Cardiovasc Interv. Author manuscript; Available in PMC 2020 Jun 1. (PMC6557424; doi:10.1002/ccd.28179)
Supplement: Video Legends [file NIHMS1023562-supplement-Video_Legends.docx]

**Video 1**

Prior to case 1, a transesophageal echocardiogram revealed severe aortic stenosis with a mean gradient of 49 mmHg, peak velocity of 4.47 m/s, and an aortic valve area of 0.73 cm2, as well as moderate aortic insufficiency, mild‐to‐moderate mitral regurgitation, and mild‐to‐moderate tricuspid regurgitation.

**Video 2**

In case 1, aortotomy revealed a severely calcified aortic bioprosthesis embedded in the surrounding aortic wall and coronary ostia. The native valve leaflets and posts were excised, and a #23 Medtronic Evolut valve (Minneapolis, MN) was inserted and deployed within the pre‐existing sewing ring. The aortotomy was closed and patched with bovine pericardium.

**Video 3**

Following case 1, a transthoracic echocardiogram from postoperative day #6 revealed adequate aortic bioprosthesis function, with trace paravalvular regurgitation. Aortic valve peak and mean gradients were 46.5 mmHg and 25.5 mmHg, respectively, and the aortic valve peak velocity was 3.4 m/s. The aortic valve area measured 1.3 cm2 with an aortic valve area index of 0.9 cm2/m2.

**Video 4**

Following case 2, a transthoracic echocardiogram from postoperative day #9 revealed adequate mitral bioprosthesis function, with trace mitral regurgitation.
